# Association of Plasma Vitamin B6 With Coronary Heart Disease in Patients Undergoing Diagnostic Coronary Angiography: New Insight on Sex Differences

**DOI:** 10.3389/fcvm.2021.789669

**Published:** 2021-12-15

**Authors:** Lihua Hu, Yuxi Li, Zhihao Liu, Fangfang Fan, Benjamin Xu, Richard Xu, Yun Song, Ping Chen, Yaping Wei, Jia Jia, Long Zhang, Haoyu Weng, Qiuping Shi, Nan Zhang, Xingang Wang, Bo Zheng, Yan Zhang, Jianping Li

**Affiliations:** ^1^Department of Cardiology, Peking University First Hospital, Beijing, China; ^2^Department of Epidemiology, Harvard T.H. Chan School of Public Health, Boston, MA, United States; ^3^Department of Biostatistics, Johns Hopkins Bloomberg School of Public Health, Baltimore, MD, United States; ^4^Beijing Advanced Innovation Center for Food Nutrition and Human Health, College of Food Science and Nutritional Engineering, China Agricultural University, Beijing, China; ^5^Department of Medicine, Shenzhen Evergreen Medical Institute, Shenzhen, China; ^6^School of Pharmacy, Jinan University, Guangzhou, China; ^7^Key Laboratory of Precision Nutrition and Food Quality, Ministry of Education, Department of Nutrition and Health, College of Food Sciences and Nutritional Engineering, China Agricultural University, Beijing, China; ^8^Key Laboratory of Molecular Cardiovascular Science of Ministry of Education, Peking University, Beijing, China; ^9^NHC Key Laboratory of Cardiovascular Molecular Biology and Regulatory Peptides, Beijing, China

**Keywords:** coronary heart disease, vitamin B6, pyridoxal 5′-phosphate, sex differences, case control

## Abstract

**Aim:** To date, findings on the overall and sex-specific effects of plasma pyridoxal 5′-phosphate (PLP, active coenzyme form of vitamin B6) on the risk of coronary heart disease (CHD) have been inconsistent. This study sought to advance our understanding on the association of plasma PLP with risk of CHD, with particular attention paid to sex differences and effect modifiers.

**Methods:** We conducted a hospital-based, case-control study on suspected CHD patients undergoing diagnostic coronary angiography. A total of 429 CHD cases and 429 controls matched by age, sex, and operation time were included in the final analysis. Plasma PLP was assessed using LC-MS. Logistic regression analyses were performed to evaluate the association between plasma PLP and a first CHD event.

**Results:** The mean (SD) plasma PLP levels were 8.4 (6.3) in male cases and 9.0 (11.0) in female cases, and 9.5 (8.5) in male controls and 12.5 (12.9) in female controls. Each 1 ng/mL increment in log_2_PLP was associated with a 28% lower risk of CHD in overall population. When stratified by sex, plasma PLP was significantly and independently associated with CHD in women (OR = 0.63, 95% CI: 0.50–0.80), but not in men (OR = 0.86, 95% CI: 0.67–1.09). The association of plasma PLP with CHD risk was modified by sex (adjusted *P*_interaction_ = 0.022).

**Conclusions:** We found a significant, inverse linear association between plasma PLP and CHD in Chinese women, but not in men. Our findings warrant additional investigation.

## Introduction

Cardiovascular diseases (CVDs) are the leading cause of global disease burden and a major contributor to disability (1, 2). Worldwide, CVDs caused 18.6 million deaths in 2019. Among CVDs, 49% were attributable to coronary heart disease (CHD) (1). Epidemiological investigations show that the age for developing CHD is becoming younger (3). CHD is a major cause of both death and disability in developed countries and is responsible for one-third or more of all deaths in individuals over the age of 35 (4). The etiology and pathogenesis of CHD are not fully understood. Early identification and treatment of individuals at risk for CHD are essential for decreasing cardiovascular morbidity and mortality (4). Despite significant efforts to reduce well-known traditional risk factors (e.g., hypertension, diabetes, dyslipidemia, abdominal obesity, and smoking), many countries, including China, continue to observe rising CHD incidence and mortality. It underscores the need to identify new risk factors to minimize residual risk. It is estimated that 20% of CHD in the US is attributable to poor diet quality, including low intake of fruit, vegetables, dairy products, and whole grains (5). Thus, the potential role of dietary factors has gained considerable attention, particularly vitamin B6 deficiency (6–8).

Vitamin B6 (B6) is an essential micronutrient. There is increasing interest in B6 as a risk factor for CHD because of its involvement in one-carbon metabolism, the metabolism of carbohydrates, amino acids, lipids, neurotransmitters, and gluconeogenesis and glycogenolysis (9). As a coenzyme, B6 is involved in over 160 different biochemical processes. Plasma pyridoxal 5'-phosphate (PLP) is the active coenzyme form of B6 and currently the most frequently used biomarker of B6 status (9). It has been reported that B6 deficiency is common in patients with CHD (10, 11). Gvosdova et al. (12) found that PLP levels were markedly decreased in CHD patients. Therefore, a better understanding of the role of B6 in CHD may help identify new venues to reduce the huge burden of CHD.

To date, studies on the association between B6 and CHD have yielded contradictory results. Several studies reported that low B6 was associated with increased risk of CHD (13–16). Other studies showed that B6 had no effect on risk of CHD (17, 18). Notably, for most studies, B6 was evaluated through an assessment of dietary intake, rather than blood plasma levels, which presents inherent limitations. Measurement bias might occur when measuring B6 intake, and dietary intake of B6 does not accurately represent biological dose. Moreover, diagnosis of CHD for these studies was based on questionnaire surveys, which introduces recall bias. To date, evidence regarding the association of plasma B6 with CHD in Chinese populations is limited. Furthermore, the nature of the dose-response relation is not yet known. Additionally, few previous studies have comprehensively investigated the potential modifiers of the association between B6 and CHD. In particular, it is well known that men and women differ significantly in PLP levels and CVD risk factors. Whether there is sex difference in the B6-CHD association remains to be investigated.

To address these knowledge gaps, we examined the B6-CHD association among Chinese adults. We were particularly interested in testing the hypothesis that the association between plasma PLP and CHD can be modified by sex. Findings from this study may help inform new venues for more precise prediction and prevention of CHD.

## Materials and Methods

### Study Design and Population

This observational study recruited consecutive consenting individuals aged ≥18 years with suspected CHD undergoing coronary angiography at the Department of Cardiology, Peking University First Hospital. Information on demographics, lifestyle factors, medical history, and disease status was collected through an electronic medical record system. Blood was drawn at the time of angiograph. Inclusion criteria were men and women aged ≥18 years with suspected CHD undergoing coronary angiography. Exclusion criteria were as follows: a history of physician diagnosed CHD; serum cardiac troponin (cTnI) ≥0.04 ng/mL; inability to provide informed consent; refusal to supply a venous blood sample. The study was approved by the ethics review board of Peking University First Hospital (Beijing, China). All participants provided written informed consent.

**Figure 1:**
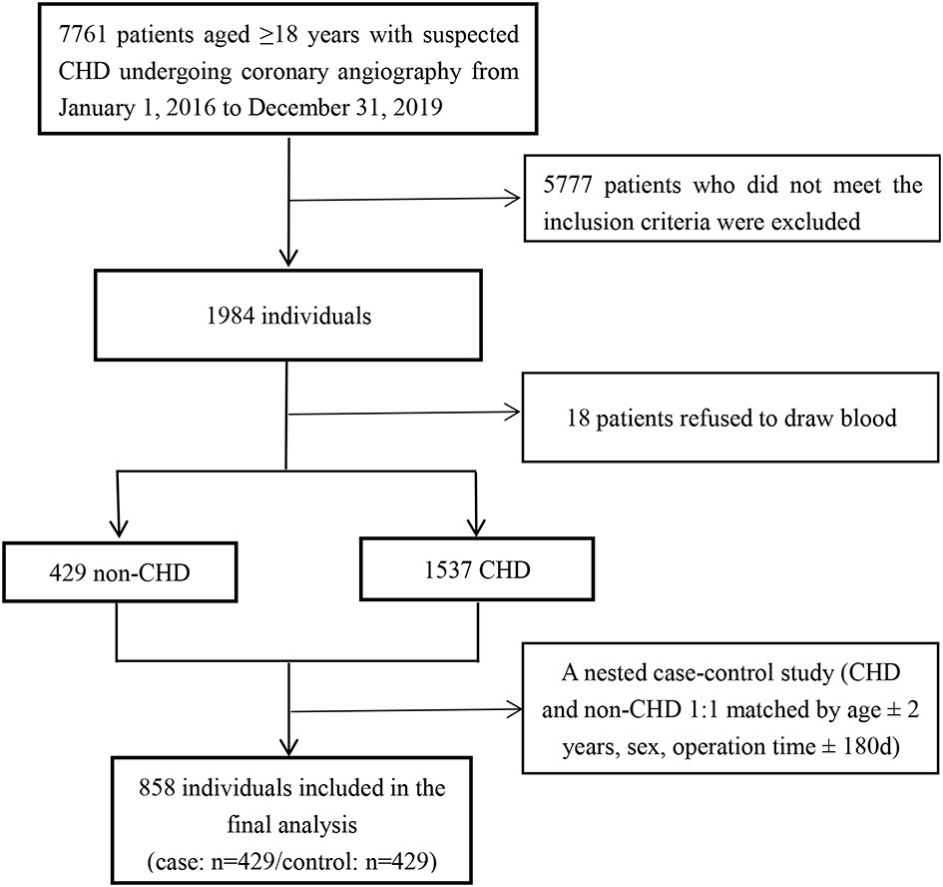
Flow chart of study participants.

Figure 1 is the flowchart of study participants. From January 1, 2016, to December 31, 2019, 7,761 patients were screened. Of these patients, 1984 were determined to be eligible for the study based on the inclusion and exclusion criteria. Patients without a blood sample for vitamin detection (*n* = 18) were excluded, leaving a final total of 1,966 eligible participants. A hospital-based, case-control cohort within this population was established. Cases were defined as having at least 70% stenosis of one major coronary artery by coronary angiography. Control subjects were chosen from those who remained free of CHD, determined by cardiac catheterization, and defined as having <30% stenosis of all major coronary arteries. We identified 1,537 incident CHD cases and 429 non-CHD controls. Cases and controls were matched on age (within 2 years), sex, and operation time (within 3 months) on a 1:1 ratio. Thus, the sample for analysis consisted of 429 matched pairs of cases and controls.

### Laboratory Measurements of Plasma PLP

Blood was drawn at the time of angiograph. B6 was measured in the form of plasma PLP by liquid chromatography-tandem mass spectrometry (LC-MS). The limit of detection (LOD) for plasma PLP was 1 ng/mL. The average recovery rate of extraction was above 90%. Both intra-assay and inter-assay CVs of duplicate samples (randomly placed among the study samples) were calculated as <15%.

### Covariates

For this study, information on patient demographics, lifestyle, family history, comorbidities, medication use, and CVD biomarker variables were collected, and served as covariates in the analyses. Demographic variables included age, sex, and education. Lifestyle variables included smoking and drinking status, and body mass index (BMI). Physician-diagnosed hypertension, diabetes or hyperlipidemia were considered as covariates since they are common comorbidities for CVD. Information on family history of CHD was also collected. The CVD biomarkers used in this study included systolic blood pressure (SBP), diastolic blood pressure (DBP), fasting plasma glucose (FBG), homocysteine (hcy) and low-density lipoprotein cholesterol (LDL-C). Body mass index (BMI) was calculated as body weight in kilograms divided by the square of height in meters (kg/m^2^). Any other variables thought to be confounders based on existing literature and clinical judgment were also included in the analysis. The following variables were used in the fully adjusted model: continuous variables included age (years), BMI (kg/m^2^), and hcy (μmol/L); categorical variables included sex (male, female), smoking status (never, ever, current), drinking status (never, ever, current), hypertension (no, yes), diabetes (no, yes), hyperlipidemia (no, yes) and family history of CHD (no, yes).

### Definition of Hypertension, Diabetes, and Hyperlipidemia

Hypertension was defined by ≥1 of the following criteria: SBP ≥ 140 mmHg or DBP ≥ 90 mmHg or self-reported physician diagnosis of hypertension or usage of antihypertensive drugs (19). Diabetes was defined as self-reported physician-diagnosed diabetes or a FBG >126 mg/dL. We defined diabetes by the presence of any one of the following 5 criteria: previous diagnosis of diabetes, intake of antidiabetic medications or insulin, glycated hemoglobin level of ≥6.5%, FBG level of ≥126 mg/dL (≥7.0 mmol/L), or a 2-h glucose level of ≥200 mg/dL (≥11.1 mmol/L) after an oral glucose tolerance test (20). Participants who did not satisfy any of these 5 criteria were defined as without diabetes. Hyperlipidemia was defined as self-reported physician-diagnosed hyperlipidemia or usage of lipid-lowering drugs.

### Statistical Analysis

Continuous data are presented as mean ± standard deviation for normally distributed variables, and as median (interquartile range) for skewed distribution variables. Categorical variables are presented as number and percentage. Differences in baseline characteristics between CHD cases and controls were compared using *t*-tests for continuous variables and χ^2^ tests for categorical variables. The population characteristics by PLP tertiles were compared using ANOVA tests (continuous variables), or χ^2^ tests (categorical variables), accordingly. Because the distribution of values for PLP was strongly skewed toward the upper end, PLP was log_2_-transformed for analysis. The association between plasma PLP and CHD was examined as a continuous variable per 1 ng/mL increment in log_2_PLP and also as a categorical variable using tertiles with tertile 1 (T1) as the reference group. Taking plasma PLP as the independent variable and CHD as the dependent variable, logistic regression analysis was performed to calculate the odds ratios (ORs) and 95% confidence intervals (CIs), without and with adjustment for sex (only for total population), age, BMI, smoking status, drinking status, hypertension, diabetes, hyperlipidemia, family history of CHD, and hcy. To further address the shape of the doseresponse relation of plasma PLP and CHD, a cubic spline function model and smooth curve (penalized spline method) were fitted.

To evaluate the potential effect modification, stratified analyses were further assessed for age (<65, ≥65 years), BMI (<25, ≥25 kg/m^2^), smoking status (never, ever+current), drinking status (never, ever+current), hypertension (no, yes), diabetes (no, yes), hyperlipidemia (no, yes), family history of CHD (no, yes), and hcy (<15, ≥15 μmol/L). Heterogeneity across subgroups was assessed by fitting simultaneous logistic regressions and are presented in forest plots. Interactions between subgroups and plasma PLP were examined by likelihood ratio testing.

A series of sensitivity analyses were performed to test the robustness of the results. First, the population characteristics by plasma PLP tertiles in men and women were compared. Second, dummy variables were used to indicate missing covariate values. Second, we imputed the median values for continuous variables (i.e., BMI and hcy) and used a missing indicator approach for categorical variables (i.e., smoking, drinking and family history of CHD). The main results were also performed using the imputed datasets to minimize bias. Third, tests for linear trend were performed by entering the median value of each category of plasma PLP as a continuous variable in the models. The purpose was to verify the results of plasma PLP as a continuous variable and to observe the possibility of non-linearity.

All analyses were performed using the statistical package R (http://www.R-project.org, The R Foundation). All *P*-values were two-sided with a significance level of <0.05.

## Results

### Baseline Characteristics of the Study Participants

**Figure 2:**
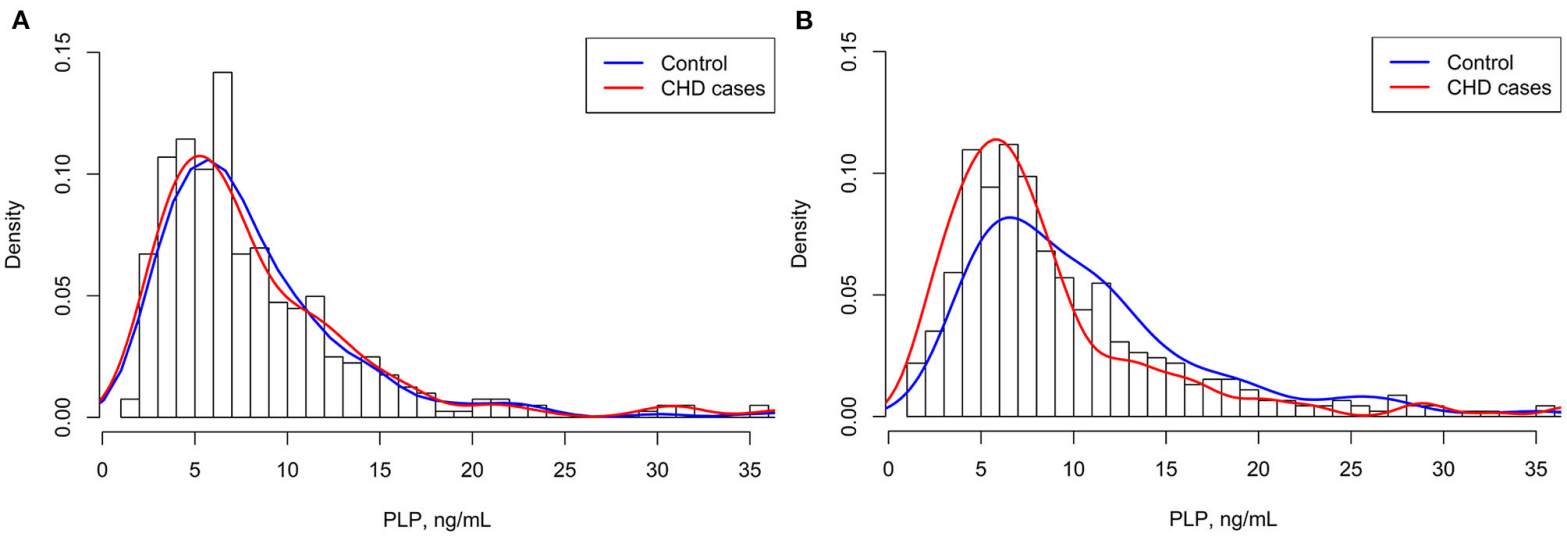
Distributions of plasma PLP in CHD cases and controls. **(A)** Men; **(B)** Women.

A total of 858 study participants (429 CHD cases and 429 matched controls) with complete PLP measurements and who met the inclusion criteria, were included in the final data analysis (Figure 1). Overall, the mean (SD) age was 63.5 (10.4) years; 46.9% were men; 69.9% were hypertensive patients; 42.1% subjects had a discharge diagnosis of diabetes; and 78.0% subjects had a discharge diagnosis of dyslipidemia. The median (IQR) duration for hypertension, diabetes and hyperlipidemia among overall population were 10.00 (15.00) years, 7.00 (11.00) years and 3.00 (9.00) year, respectively. As shown in Figure 2, the distribution of plasma PLP was skewed. The distribution of plasma PLP in the CHD cases and the controls showed heavy overlap in men (Figure 2A). There was no statistically significant difference in plasma PLP levels between CHD cases and the controls in men [6.6 (6.4) vs. 7.0 (5.4) ng/mL; *P* = 0.161]. There was a clear shift toward the right in the distribution of plasma PLP in the controls compared with the CHD cases in women (Figure 2B). Median (IQR) values of plasma PLP concentrations were lower in CHD cases [6.6 (5.1) ng/mL] than in the controls [9.2 (7.7) ng/mL; *P* < 0.001]. The median overall PLP concentration was 7.21 (interquartile range, 4.93–11.39) ng/mL.

Table 1 presents the baseline characteristics of the CHD cases and controls, stratified by sex. Among males, the CHD cases had higher rates of ever smokers, diabetes, lipid-lowering drugs and glucose-lowering drugs than those in the controls (all *P* < 0.05). Among women, compared to controls, CHD cases were more likely to be smokers, to have higher rate of hypertension, diabetes, hyperlipidemia, lipid-lowering drugs, glucose-lowering drugs, to have higher FBG levels and to have lower PLP levels (all *P* < 0.05).

**Table 1:** Characteristics of cases and control participants.

**Characteristics^a^**	**Male**			**Female**		
	**Non-CHD controls**	**CHD cases**	***P*** **-value**	**Non-CHD controls**	**CHD cases**	***P*** **-value**
*N*	201	201		228	228	
Age, years	59.9 ± 11.3	60.3 ± 11.5	0.753	65.8 ± 8.3	67.1 ± 8.3	0.110
BMI, kg/m^2^	26.4 ± 3.5	25.9 ± 3.3	0.097	26.0 ± 3.9	26.0 ± 3.9	0.988
SBP, mm Hg	132.1 ± 15.5	131.6 ± 16.1	0.768	133.3 ± 16.5	135.1 ± 16.9	0.251
DBP, mm Hg	76.9 ± 13.0	76.1 ± 10.2	0.495	73.3 ± 10.0	72.6 ± 10.3	0.449
Smoking status, *n* (%)			0.033			<0.001
Never	57 (29.2)	37 (19.2)		211 (95.9)	185 (85.6)	
Ever	61 (31.3)	80 (41.5)		1 (0.5)	11 (5.1)	
Current	77 (39.5)	76 (39.4)		8 (3.6)	20 (9.3)	
Drinking status, *n* (%)			0.940			0.675
Never	81 (41.3)	82 (42.9)		216 (96.4)	208 (95.0)	
Ever	39 (19.9)	38 (19.9)		3 (1.3)	3 (1.4)	
Current	76 (38.8)	71 (37.2)		5 (2.2)	8 (3.7)	
**Comorbidities**, ***N*** **(%)**						
Hypertension, *n* (%)	125 (62.2)	142 (70.6)	0.073	155 (68.0)	178 (78.1)	0.015
Diabetes, *n* (%)	64 (31.8)	85 (42.3)	0.030	75 (32.9)	137 (60.1)	<0.001
Hyperlipidemia, *n* (%)	150 (74.6)	165 (82.1)	0.069	167 (73.2)	187 (82.0)	0.025
Family history of CHD, *n* (%)	60 (32.6)	75 (38.9)	0.206	80 (38.3)	79 (37.6)	0.89
**Medication use**, ***N*** **(%)**						
Antihypertensive drugs	98 (48.8)	108 (53.7)	0.318	128 (56.1)	142 (62.3)	0.182
Glucose-lowering drugs	41 (20.4)	63 (31.3)	0.012	56 (24.6)	103 (45.2)	<0.001
Lipid-lowering drugs	70 (34.8)	108 (53.7)	<0.001	101 (44.3)	132 (57.9)	0.004
**Laboratory results**						
FBG, mmol/L	6.4 ± 2.2	6.8 ± 2.7	0.129	6.5 ± 2.7	8.1 ± 3.9	<0.001
LDL-C, mmol/L	2.3 ± 0.8	2.2 ± 0.8	0.243	2.5 ± 0.8	2.3 ± 0.8	0.112
Hcy, μmol/L	16.4 ± 8.1	16.4 ± 8.2	0.951	13.9 ± 7.1	14.1 ± 8.6	0.804
PLP, ng/mL	9.5 ± 8.5	8.4 ± 6.3	0.160	12.5 ± 12.9	9.0 ± 11.0	0.002

*CHD, coronary heart disease; BMI, body mass index; SBP, systolic blood pressure; DBP, diastolic blood pressure; FBG, fasting plasma glucose; LDL-C, low density lipoprotein cholesterol; Hcy, homocysteine; PLP, pyridoxal 5-phosphate.*

a *Data are presented as number (%) or mean ± SD*.

Additionally, baseline characteristics of the study participants by PLP tertiles are shown in Supplementary Table 1. Plasma PLP was inversely associated with FBG concentrations and positively associated with BMI levels in men. Women with higher PLP concentrations had lower rate of diabetes and lipid-lowering drugs and had lower FBG concentrations.

### Association Between Plasma PLP and CHD

**Figure 3:**
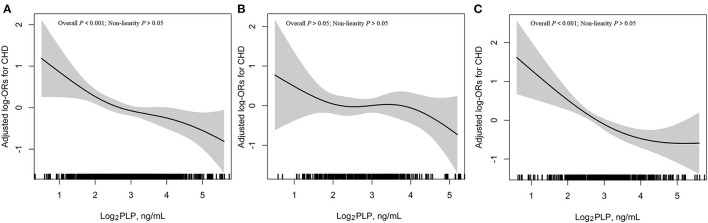
Association between plasma log_2_PLP and risk of CHD. **(A)** Overall population; **(B)** Men; **(C)** Women. Adjustment factors included sex (only for overall population), age, BMI, smoking status, drinking status, hypertension, diabetes, hyperlipidemia, family history of CHD and hcy.

The association between plasma PLP and risk of CHD based on restricted cubic splines is shown in Figure 3. Overall, there was a significant linear relation of plasma PLP with risk of CHD (*P*-linearity < 0.001) (Figure 3A). Given the differences in plasma PLP levels between male and female participants (8.9 ± 7.5 vs. 10.7 ± 12.1 ng/mL), we further investigated the possible effect of sex on the PLP-CHD association (Figures 3B,C). The results supported an inverse linear association between plasma PLP levels and risk of CHD in women (Figure 3C), but not in men (Figure 3B).

Table 2 shows the ORs of CHD in men and women. In the multivariable adjusted model (Model 2), each 1 ng/mL increment in log_2_PLP was associated with a 28% lower risk of CHD (OR = 0.72, 95% CI: 0.61–0.85). Consistently, when plasma PLP was assessed as tertiles, a significantly lower risk of CHD was found in participants in the second (OR = 0.67, 95% CI: 0.47–0.97), and third (OR = 0.50, 95% CI: 0.35–0.73) tertiles, compared with those in the first tertile. When stratified by sex, the association between plasma PLP and CHD was no longer significant among men but remained significant among women. The ORs of log_2_PLP for CHD were 0.86 (95% CI, 0.67–1.09) among men and 0.63 (95% CI, 0.50–0.80) among women, indicating a significant interaction between PLP and sex (*P* = 0.022). Analyses on tertiles of PLP yielded a similar pattern. In men, the ORs of PLP tertiles 2 and 3 for CHD were 0.55 (0.33–0.94) and 0.79 (0.46–1.35), respectively (*P* for trend = 0.379). In contrast, the ORs of the corresponding tertiles in women were 0.77 (0.46–1.28) and 0.35 (0.20–0.60), respectively (*P* for trend < 0.001). Sensitivity analysis showed that the linear trends of the data both pre- and post-imputation were approximately the same (data was not shown). The association with PLP persisted even after menopause (data was not shown). Similar associations were found after adjusting for the medication usage (Supplementary Table 2).

**Table 2:** Association between plasma PLP and risk of CHD.

**PLP, ng/mL**	**Cases/controls**	**Crude model**		**Adjusted model**	
		**OR (95% CI)**	***P*** **-value**	**OR (95% CI)**	***P*** **-value**
**Overall**					
Continuous^†^	49/429	0.70 (0.61, 0.81)	<0.001	0.72 (0.61, 0.85)	<0.001
*Tertiles*					
T1 (<5.7)	171/115	Ref.		Ref.	
T2 (5.7–9.6)	141/145	0.67 (0.48, 0.94)	0.019	0.67 (0.47, 0.97)	0.034
T3 (≥9.6)	117/169	0.47 (0.34, 0.66)	<0.001	0.50 (0.35, 0.73)	<0.001
*P* for trend		<0.001		<0.001	
**Male**					
Continuous^†^	201/201	0.86 (0.69, 1.07)	0.171	0.86 (0.67, 1.09)	0.199
*Tertiles*					
T1 (<5.4)	76/58	Ref.		Ref.	
T2 (5.4–8.9)	59/75	0.60 (0.37, 0.97)	0.038	0.55 (0.33, 0.94)	0.029
T3 (≥8.9)	66/68	0.74 (0.46, 1.20)	0.221	0.79 (0.46, 1.35)	0.389
*P* for trend		0.222		0.379	
**Female**					
Continuous^†^	228/228	0.59 (0.48, 0.72)	<0.001	0.63 (0.50, 0.80)	<0.001
*Tertiles*					
T1 (<6.1)	95/57	Ref.		Ref.	
T2 (6.1–10.3)	82/70	0.70 (0.44, 1.11)	0.1311	0.77 (0.46, 1.28)	0.313
T3 (≥10.3)	51/101	0.30 (0.19, 0.48)	<0.001	0.35 (0.20, 0.60)	<0.001
*P* for trend		<0.001		<0.001	

† *PLP value was log_2_-transformed.*

*Adjusted Model was adjusted for sex (only for overall population), age, BMI, smoking status, drinking status, hypertension, diabetes, hyperlipidemia, family history of CHD and hcy*.

### Stratified Analysis by Potential Effect Modifiers in Female Participants

**Figure 4:**
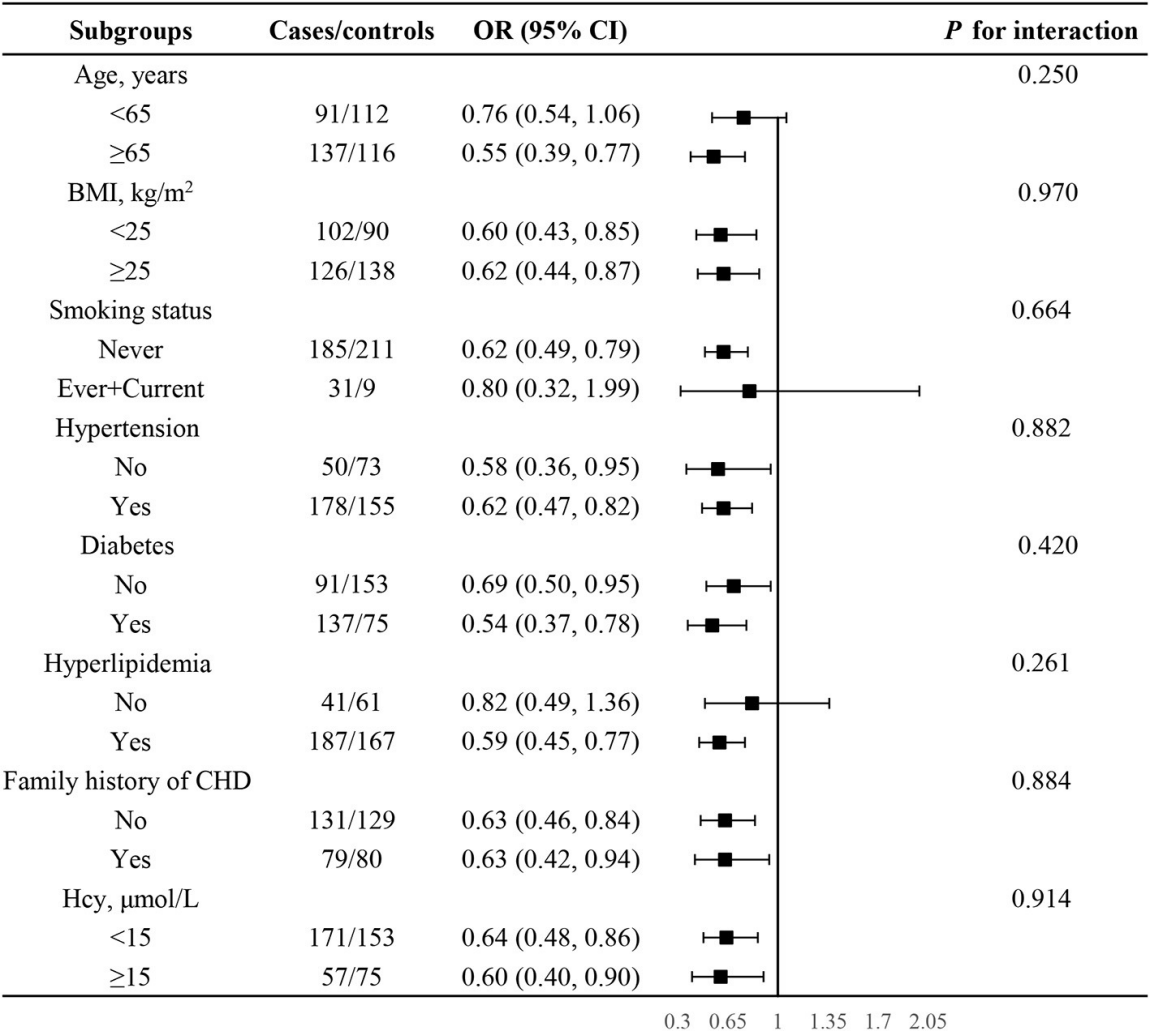
Subgroup analyses to detect effect modification on the association between plasma log_2_PLP and CHD in women. Each subgroup analysis adjusted, if not stratified, adjusted for age, BMI, smoking status, drinking status, hypertension, diabetes, hyperlipidemia, family history of CHD and hcy.

Given the significant association between PLP and CHD among women, stratified analyses were performed to further explore potential modifiers affecting the relation of log_2_PLP with risk of CHD among women (Figure 4). None of the variables, including age, BMI, smoking status, hypertension, diabetes, hyperlipidemia, family history of CHD, as well as hcy, were found to modify the association between plasma PLP and CHD (*P* for all interaction >0.05).

## Discussion

To our knowledge, this is the first Chinese hospital-based case-control study to test the hypothesis that there is an association between plasma PLP and CHD, and such an association can be modified by sex. The present study lent support for this hypothesis by demonstrating an inverse association between plasma PLP and CHD, and differential association between men and women.

B6 has received much attention following the recognition of hcy as a potential risk factor for CHD (21). However, previous studies on the association between B6 and CHD yielded conflicting results. Our study demonstrated an inverse association between plasma PLP and CHD in the overall population. Consistent with our findings, several recent studies have observed an inverse association between plasma PLP and CHD. A cross-sectional study of 700 Taiwanese participants found that low plasma PLP was independently associated with an increased risk of CHD (22). A meta-analysis that included eleven prospective cohort studies (total *n* = 369,746) with 5133 cases of CHD demonstrated a linear, inverse association between B6 intake and risk of CHD (15). However, several reports failed to identify an association between plasma PLP and risk of CHD. Dierkes et al. (23) conducted a nested case-cohort study based on 26,761 participants aged 35–65 years at baseline and found that the association between plasma PLP and CHD was abolished after adjusting for inflammation and smoking. Minovi et al. (24) used data from 6,249 participants and found that the plasma PLP-cardiovascular outcomes association was confounded by traditional risk factors and inflammation. These conflicting results might be attributed to differences in study design, sample size, populations, PLP level, and adjustment of confounders.

The current study provides several new insights. Our data showed that sex significantly modified the association of plasma PLP with CHD. We identified a significant, inverse linear association between plasma PLP and CHD in Chinese women, but not in men. Two previous population-based cohort studies analyzed the association between vitamin B6 and CVD with a particular attention to sex differences and yielded conflicting results. Minov et al. (24) found that the association of plasma PLP with risk of cardiovascular outcomes was stronger in women compared to men. In contrast, a prospective cohort study enrolling 9,142 Korean participants aged 40–69 years found that higher intake of B6 could reduced CVD risk in men, but not in women (25). Moreover, it has been described that B6 deficiency can cause atherosclerosis in female rats, but not in male rats (26). It may be attributed to the sex-specific difference in plasma PLP levels and CVD risk factors. In our study, females had higher PLP levels compared to males. This could be related to differences in how plasma PLP is metabolized between the sexes (27). Moreover, in our study, CHD risk factors in men and women were different. Males were more likely to have lower values for age, SBP, LDL-C and PLP, to have higher values for hcy, to be smokers and drinkers, and to have lower rates of hypertension, diabetes and medication use than those of females (the data was not shown in our paper). Thus, the effect modification of sex may be attributed to the complex interactions between vitamin B6 and CHD risk factors. The exact mechanism underlying the sex-dependent effect of vitamin B6 on CHD risk is still unclear. Additional research is warranted.

Vitamin B6 has several cardioprotective features that could explain these findings. B6 has anti-inflammatory properties (28), helps the antioxidant defense system fight against oxidative stress (29), and has some important functions within the immune system (30). Also, vitamin B6 is involved in the one-carbon unit's metabolic pathways, and thereby plays important roles in DNA methylation and repair (31). Vitamin B6 contributes to the transsulfuration pathway of hcy metabolism which is associated with a higher risk of developing CHD (32). Recent studies have revealed that vitamin B6 treatment increases cardiac levels of imidazole dipeptides (e.g., carnosine, anserine, and homocarnosine), histamine, and γ-aminobutyric acid and suppresses P2X7 receptor-mediated NLRP3 inflammasome. These modulations may imply potential cardioprotective mechanisms of vitamin B6 (33). Furthermore, vitamin B6 has been shown to inhibit platelet aggregation and endothelial cell proliferation, which suggests an antithrombotic effect (34). However, the exact mechanism underlying B6-CHD association remains to be studied by *in vitro* and *in vivo* experiments.

Our study findings have important clinical implications. Data from the present study show a sex-difference in the association between plasma PLP concentration and CHD. The inverse association existed among women but not in men. Hence, our findings suggest we should take sex differences into account and stratify data by sex when evaluating B6 and CHD association in the future. If further confirmed, our findings may provide important data for clinical and nutritional guidelines on the primary prevention of CHD among women, where plasma PLP may serve as a potentially modifiable factor and a possible biomarker for the purposes of monitoring and intervention to prevent CHD. Furthermore, one future direction of this work is to clarify the potential mechanisms of sex disparities in plasma PLP and CHD.

There are several key limitations with the present study. First, as in all observational studies, we could not exclude residual confounding from unmeasured factors even though known potential confounding factors were controlled for. Second, we cannot draw a causal conclusion from the present results. Third, we did not have detailed food intake information. Forth, because it was a single-center observational study, the generalizability of our results to other populations remains to be verified. Despite these limitations, our research also has several strengths. First, we measured plasma PLP, which is the bioactive form of circulating B6. Second, both CHD cases and controls were determined by the coronary angiography, a standard method for clinical diagnosis of CHD. Third, we explored and found a significant sex-PLP interaction on CHD in a Chinese population. Finally, in females, we conducted sensitivity analyses to examine PLP-CHD association across various subgroups and found robust results.

In summary, this hospital-based case-control study showed a significant linear, inverse association between plasma PLP and CHD in Chinese women, but not in men. Further longitudinal investigations are needed to confirm our findings and to elucidate their mechanisms. Moreover, the findings underscore the need for further research to establish an optimal range of plasma PLP in the Chinese men and women, respectively. Doing so would provide more tailored clinical and nutritional guidelines on optimal PLP levels for the primary prevention of CHD.

## Data Availability Statement

The raw data supporting the conclusions of this article will be made available by the authors, without undue reservation.

## Ethics Statement

The study protocol was approved by the Ethics Review Board of Peking University First Hospital (Beijing, China). The patients/participants provided their written informed consent to participate in this study.

## Author Contributions

JL: conceptualization, resources, visualization, and supervision. YS, PC, and JL: methodology. LH, FF, and YS: software. BX, RX, and YZ: validation. LH and YW: formal analysis. YL, LZ, HW, QS, NZ, XW, and BZ: investigation. ZL and JJ: data curation. LH: writing—original draft preparation. BX, RX, and JL: writing—review and editing. YZ and JL: project administration. LH and JL: funding acquisition. All authors have read and agreed to the published version of the manuscript.

## Funding

The study was supported by Peking University Medicine Fund of Fostering Young Scholars' Scientific and Technological Innovation (Grant 34254), Projects of National Natural Science Foundation of China (Grant 82070458), Capital's Funds for Health Improvement and Research (Grant 2020-2Z-40714), and Beijing Municipal Science and Technology Project (Grant Z191100006619039).

## Conflict of Interest

The authors declare that the research was conducted in the absence of any commercial or financial relationships that could be construed as a potential conflict of interest.

## Publisher's Note

All claims expressed in this article are solely those of the authors and do not necessarily represent those of their affiliated organizations, or those of the publisher, the editors and the reviewers. Any product that may be evaluated in this article, or claim that may be made by its manufacturer, is not guaranteed or endorsed by the publisher.
