# Supplementary material for: Association of Plasma Vitamin B6 With Coronary Heart Disease in Patients Undergoing Diagnostic Coronary Angiography: New Insight on Sex Differences
Source: Front Cardiovasc Med. 2021 Dec 15;8:789669. doi: 10.3389/fcvm.2021.789669 (PMC8714911; doi:10.3389/fcvm.2021.789669)
Supplement: Supplementary file 1 [file Table_1.DOCX]

Supplementary Material

**Table S1. Characteristics of study population based on tertiles of plasma PLP stratify by sexes.**

| Characteristics^*^ | Male | | |  | Female | | |  |
| --- | --- | --- | --- | --- | --- | --- | --- | --- |
|  | Plasma PLP, ng/mL | | | *P* value | Plasma PLP, ng/mL | | | *P* value |
|  | T1  (<5.4) | T2  (5.4-8.9) | T3  (≥8.9) |  | T1  (<6.1) | T2  (6.1-10.3) | T3  (≥10.3) |  |
| N | 134 | 134 | 134 |  | 152 | 152 | 152 |  |
| Age, years | 61.6±11.1 | 58.8±11.4 | 59.9±11.6 | 0.112 | 67.2±7.9 | 66.2±8.2 | 66.0±8.8 | 0.415 |
| BMI, kg/m^2^ | 25.4±3.5 | 26.2±3.2 | 26.8±3.4 | 0.003 | 26.4±4.2 | 26.1±3.7 | 25.5±3.9 | 0.161 |
| SBP, mm Hg | 132.8±16.0 | 130.4±15.1 | 132.2±16.3 | 0.451 | 135.3±16.3 | 134.6±17.9 | 132.8±15.8 | 0.415 |
| DBP, mm Hg | 75.2±12.6 | 77.9±11.4 | 76.3±10.9 | 0.173 | 73.2±10.7 | 73.5±10.5 | 72.2±9.2 | 0.503 |
| Smoking status, n (%) |  |  |  | 0.063 |  |  |  | 0.084 |
| Never | 25 (19.1) | 28 (21.7) | 41 (32.0) |  | 128 (87.7) | 132 (90.4) | 136 (94.4) |  |
| Ever | 51 (38.9) | 43 (33.3) | 47 (36.7) |  | 8 (5.5) | 2 (1.4) | 2 (1.4) |  |
| Current | 55 (42.0) | 58 (45.0) | 40 (31.2) |  | 10 (6.8) | 12 (8.2) | 6 (4.2) |  |
| Drinking status, n (%) |  |  |  | 0.914 |  |  |  | 0.796 |
| Never | 55 (41.7) | 57 (44.2) | 51 (40.5) |  | 139 (94.6) | 143 (96.6) | 142 (95.9) |  |
| Ever | 29 (22.0) | 24 (18.6) | 24 (19.0) |  | 3 (2.0) | 2 (1.4) | 1 (0.7) |  |
| Current | 48 (36.4) | 48 (37.2) | 51 (40.5) |  | 5 (3.4) | 3 (2.0) | 5 (3.4) |  |
| **Comorbidities,** N (%) |  |  |  |  |  |  |  |  |
| Hypertension, n (%) | 90 (67.2) | 86 (64.2) | 91 (67.9) | 0.791 | 114 (75.0) | 115 (75.7) | 104 (68.4) | 0.291 |
| Diabetes, n (%) | 54 (40.3) | 49 (36.6) | 46 (34.3) | 0.593 | 83 (54.6) | 70 (46.1) | 59 (38.8) | 0.022 |
| Hyperlipidemia, n (%) | 99 (73.9) | 107 (79.9) | 109 (81.3) | 0.292 | 117 (77.0) | 123 (80.9) | 114 (75.0) | 0.451 |
| Family history of CHD, n (%) | 40 (32.0) | 51 (40.2) | 44 (35.2) | 0.396 | 52 (36.9) | 55 (37.9) | 52 (39.1) | 0.931 |
| **Medication use, N (%)** |  |  |  |  |  |  |  |  |
| Antihypertensive drugs | 70 (52.2) | 69 (51.5) | 67 (50.0) | 0.933 | 91 (59.9) | 94 (61.8) | 85 (55.9) | 0.564 |
| Lipid-lowering drugs | 39 (29.1) | 30 (22.4) | 35 (26.1) | 0.453 | 65 (42.8) | 51 (33.6) | 43 (28.3) | 0.028 |
| Glucose-lowering drugs | 59 (44.0) | 62 (46.3) | 57 (42.5) | 0.826 | 80 (52.6) | 84 (55.3) | 69 (45.4) | 0.204 |
| **Laboratory results** |  |  |  |  |  |  |  |  |
| FBG, mmol/L | 7.1±2.8 | 6.5±2.6 | 6.4±2.0 | 0.046 | 8.2±4.0 | 6.8±2.8 | 6.9±3.1 | <0.001 |
| LDL-C, mmol/L | 2.2±0.7 | 2.3±0.8 | 2.3±0.8 | 0.457 | 2.4±0.9 | 2.4±0.8 | 2.4±0.9 | 0.926 |
| Hcy, μmol/L | 15.7±7.9 | 16.3±8.9 | 17.1±7.5 | 0.340 | 14.5±10.0 | 12.8±4.0 | 14.7±8.4 | 0.077 |

Abbreviations: CHD = coronary heart disease; BMI = body mass index; SBP = systolic blood pressure; DBP = diastolic blood pressure; LDL-C = low density lipoprotein cholesterol; Hcy = homocysteine; PLP = pyridoxal 5-phosphate.

^a^Data are presented as number (%) or mean±SD

**Table S2. Association between plasma PLP and risk of CHD**

| PLP, ng/mL | Cases/controls | Model 1 | | Model 2 | |
| --- | --- | --- | --- | --- | --- |
|  |  | OR (95% CI) | *P* value | OR (95% CI) | *P* value |
| Overall |  |  |  |  |  |
| Continuous^†^ | 49/429 | 0.70 (0.61, 0.81) | <0.001 | 0.72 (0.62, 0.85) | <0.001 |
| Tertiles |  |  |  |  |  |
| T1 (<5.7) | 171/115 | Ref. |  | Ref. |  |
| T2 (5.7-9.6) | 141/145 | 0.67 (0.48, 0.94) | 0.019 | 0.69 (0.48, 1.00) | 0.047 |
| T3 (≥9.6) | 117/169 | 0.47 (0.34, 0.66) | <0.001 | 0.51 (0.35, 0.74) | <0.001 |
| *P* for trend |  | <0.001 |  | <0.001 |  |
| Male |  |  |  |  |  |
| Continuous^†^ | 201/201 | 0.86 (0.69, 1.07) | 0.171 | 0.84 (0.66, 1.07) | 0.163 |
| Tertiles |  |  |  |  |  |
| T1 (<5.4) | 76/58 | Ref. |  | Ref. |  |
| T2 (5.4-8.9) | 59/75 | 0.60 (0.37, 0.97) | 0.038 | 0.56 (0.33, 0.96) | 0.035 |
| T3 (≥8.9) | 66/68 | 0.74 (0.46, 1.20) | 0.221 | 0.78 (0.45, 1.36) | 0.385 |
| *P* for trend |  | 0.222 |  | 0.361 |  |
| Female |  |  |  |  |  |
| Continuous^†^ | 228/228 | 0.59 (0.48, 0.72) | <0.001 | 0.64 (0.51, 0.81) | <0.001 |
| Tertiles |  |  |  |  |  |
| T1 (<6.1) | 95/57 | Ref. |  | Ref. |  |
| T2 (6.1-10.3) | 82/70 | 0.70 (0.44, 1.11) | 0.1311 | 0.76 (0.45, 1.26) | 0.286 |
| T3 (≥10.3) | 51/101 | 0.30 (0.19, 0.48) | <0.001 | 0.36 (0.21, 0.61) | <0.001 |
| *P* for trend |  | <0.001 |  | <0.001 |  |

^†^PLP value was log_2_-transformed.

Model 1: no covariates were adjusted.

Model 2 was adjusted for adjusted for sex (only for overall population), age, BMI, smoking status, drinking status, hypertension, diabetes, hyperlipidemia, family history of CHD, hcy, **antihypertensive drugs, glucose-lowering drugs, and lipoprotein-lowering drugs**.
